# Gene mobility promotes the spread of resistance in bacterial populations

**DOI:** 10.1038/ismej.2017.42

**Published:** 2017-03-31

**Authors:** Cagla Stevenson, James PJ Hall, Ellie Harrison, AJamie Wood, Michael A Brockhurst

**Affiliations:** 1 Department of Biology, University of York, York Y010 5DD, UK; 2 Department of Mathematics, University of York, York Y010 5DD, UK

## Abstract

Theory predicts that horizontal gene transfer (HGT) expands the selective conditions under which genes spread in bacterial populations. Whereas vertically inherited genes can only spread by positively selected clonal expansion, mobile genetic elements can drive fixation of genes by infectious HGT. We tested this using populations of *Pseudomonas fluorescens* and the conjugative mercury resistance (Hg^R^) plasmid pQBR57. HGT expanded the selective conditions allowing the spread of Hg^R^: Chromosomal Hg^R^ only increased in frequency under positive selection, whereas plasmid-encoded Hg^R^ reached fixation with or without positive selection. Tracking plasmid dynamics over time revealed that the mode of Hg^R^ inheritance varied across mercury environments. Under mercury selection, the spread of Hg^R^ was driven primarily by clonal expansion while in the absence of mercury Hg^R^ dynamics were dominated by infectious transfer. Thus, HGT is most likely to drive the spread of resistance genes in environments where resistance is useless.

Microbial populations reproduce clonally by vertical descent but can also exchange genes by horizontal gene transfer (HGT). HGT is an important process in bacterial evolution, accelerating adaptation by allowing the spread of ecologically and clinically relevant traits between lineages (Frost *et al.*, 2005; Thomas and Nielsen, 2005). Therefore, the balance of vertical versus horizontal inheritance is expected to have important effects on bacterial evolution and thus function (Smith *et al.*, 1993; Cordero and Polz, 2014; Shapiro, 2016). Comparative genomics has revealed that bacterial species undergo dramatic shifts in the balance of vertical versus horizontal inheritance over time (Cordero and Polz, 2014). These shifts may be due to changes in selection on inherited traits, as theory predicts that vertical inheritance is favoured by strong positive selection, increasing clonality via genome-wide selective sweeps (clonal expansion), whereas horizontally inherited genes can spread even in the absence of positive selection (Tazzyman and Bonhoeffer, 2014), maintaining population genomic diversity. However, experimental data addressing this issue are lacking.

To test how selection alters the balance of vertical versus horizontal transmission of bacterial genes, we quantified the dynamics of mercury resistance (Hg^R^) in populations of the soil bacterium *Pseudomonas fluorescens* SBW25 (Rainey and Bailey, 1996) with the Hg^R^ operon *mer* encoded either chromosomally or carried on an Hg^R^ plasmid pQBR57 (Lilley and Bailey, 1997). We established 36 replicate populations of SBW25, 18 with Hg^R^ encoded on their chromosome (non-horizontally transferable), 18 with Hg^R^ encoded on pQBR57 (horizontally transferable). Each population was mixed 50:50 with a mercury-sensitive differentially marked SBW25 strain and then propagated by serial transfer every 24 h for 8 days. Populations were grown in one of three mercury environments, 0, 20 and 40 μM HgCl_2_ (six replicates per treatment); this represents a selective gradient wherein plasmid-encoded Hg^R^ is under, respectively, strong negative selection, weak positive selection and strong positive selection, due to the balance between the cost of plasmid carriage and the benefits of Hg^R^ (Supplementary Figure S1; Hall *et al.*, 2015). Because pQBR57 is maintained at low copy number (Hall *et al.*, 2015), the chromosomal and plasmid-encoded Hg^R^ genes provide equivalent levels of resistance (Supplementary Figure S2). Every 2 days we determined the proportion of Hg^R^ cells within each population by plate counts. Furthermore, as our donors and recipients were differentially marked we were able to track the frequency of pQBR57 in both donor and recipient populations (for full methods see Supplementary Information).

The end point proportion of mercury-resistant (Hg^R^) cells in the population was significantly affected by the horizontal transmissibility of Hg^R^ (Figure 1; main effect of mobility at 0 μM HgCl_2_: *F*_1,10_=74.34, *P*<0.001). Where Hg^R^ was encoded on the chromosome, positive selection was required to drive the spread of resistance: Hg^R^ rapidly became fixed within the population in the 20 and 40 μM HgCl_2_ environments, whereas in the 0 μM HgCl_2_ environment chromosomal Hg^R^ remained at ~50% prevalence. In contrast, when Hg^R^ was encoded on the conjugative plasmid pQBR57, and thus horizontally transferable, Hg^R^ reached high frequencies across all mercury environments (0, 20 and 40 μM HgCl_2_). Thus, the opportunity for horizontal transfer expanded the selective conditions allowing the fixation of Hg^R^ such that this occurred both with and without positive selection for resistance.

Tracking plasmid dynamics over time revealed that the strength of positive selection determined the balance of horizontal versus vertical inheritance of plasmid-encoded Hg^R^ in bacterial populations. HGT played a significantly greater role as the strength of selection decreased (Figure 2; Main effect of mercury: *F*_1,16_=392.72, *P*<0.001). Under strong positive selection (40 μM HgCl_2_), Hg^R^ swept through the population by clonal expansion of the original Hg^R^ donor population. This was presumably due to the high toxicity of the environment strongly selecting against plasmid-free recipients, limiting the opportunity for HGT via plasmid conjugation as a consequence. The contribution of vertical inheritance to the spread of Hg^R^ reduced with weakening positive selection. Under weak positive selection (20 μM HgCl_2_), Hg^R^ spread through the population by a mixture of vertical clonal expansion of donor cells and horizontal transmission of the plasmid into the recipient subpopulation. Under negative selection (0 μM HgCl_2_) Hg^R^ spread by conjugative plasmid transfer into available plasmid-free recipient cells. Therefore, while strong positive selection favoured vertical inheritance, the contribution of horizontal transfer to the spread of resistance genes increased as positive selection weakened.

Our data are consistent with theory that HGT can overcome selective barriers to drive the spread of resistance genes in the absence of positive selection, whereas resistance genes spread through vertical transmission only under positive selection. Thus, whereas positive selection for resistance would purge genomic diversity via genome-wide sweeps of resistance (Wiedenbeck and Cohan, 2011), negative selection against resistance coupled with infectious HGT of resistance genes can spread resistance genes into diverse genomic backgrounds. Consequently, the sharing of resistance genes between lineages is most likely to occur in environments without positive selection, and therefore where resistance genes have little use.

## Figures and Tables

**Figure 1:**
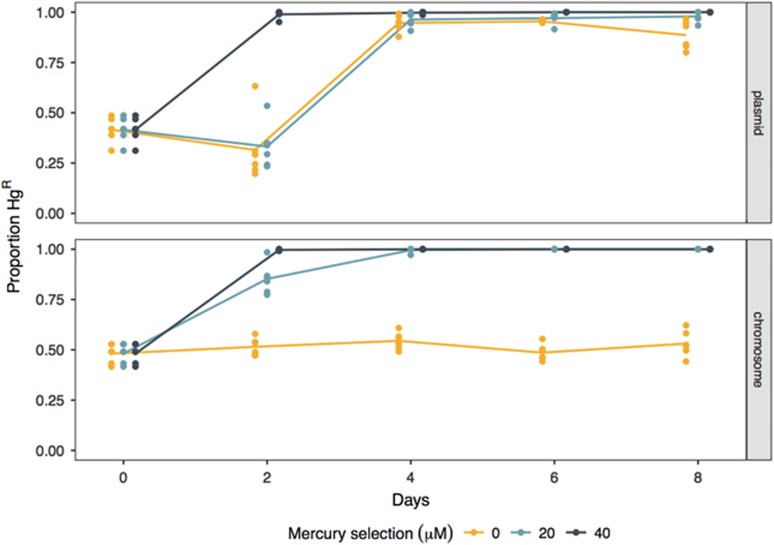
Horizontal transmission had a significant impact on the proportion of Hg^R^. The proportion of chromosome- and plasmid-encoded Hg^R^ was determined over time across the three mercury treatments (0, 20 and 40 μM HgCl_2_). Points represent replicate populations and are slightly offset by treatment on the *x* axis to prevent over plotting. Lines represent means (*n*=6).

**Figure 2:**
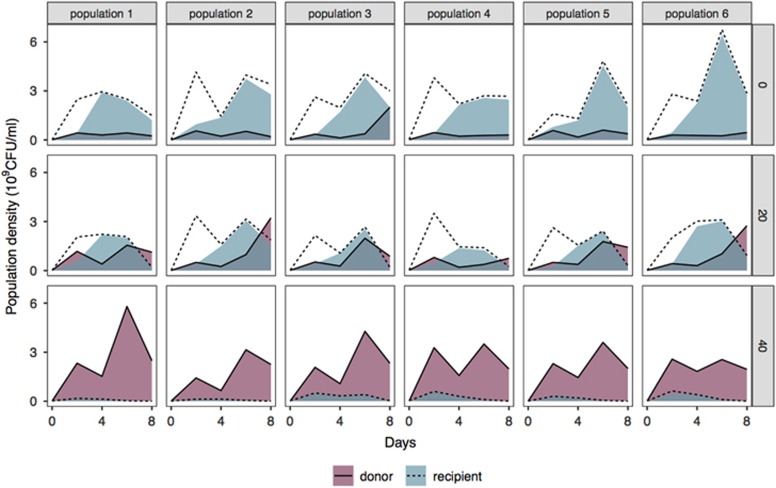
Selection determines the balance of horizontal versus vertical inheritance of plasmid-encoded Hg^R^. Plasmid transfer in each of six replicate populations was tracked over time across the three mercury treatments (0, 20 and 40 μM HgCl_2_). Dotted lines indicate densities of recipient populations; solid lines indicate densities of donor populations. For each population, shaded regions represent plasmid prevalence within donor (purple) and recipient (blue) subpopulations.
