# Supplementary material for: Gene mobility promotes the spread of resistance in bacterial populations
Source: ISME J. 2017 Mar 31;11(8):1930–2. doi: 10.1038/ismej.2017.42 (PMC5496671; doi:10.1038/ismej.2017.42)
Supplement: Supplementary Information [file ismej201742x1.docx]

**SUPPLEMENTARY INFORMATION**

**METHODS**

*Strains and culture conditions*

Experimental populations were founded using *P. fluorescens* SBW25 (Rainey and Bailey, 1996). Initially, isogenic strains were constructed with mobile or non-mobile Hg^R^. An Hg^R^ strain, harbouring the resistance transposon Tn5042 on the bacterial chromosome, was used to construct a Hg^S^ control strain using protocols for site directed mutagenesis (Heckman and Pease, 2007). Following this the plasmid pQBR57 (Lilley and Bailey, 1997) was then conjugated into this strain using standard protocols (Simonsen *et al.*, 1990; Hall *et al.*, 2015). Hg^R^ and Hg^S^ populations were labelled with mini-Tn7 gentamicin resistance (Gm^R^) and streptomycin resistance lacZ (Sm^R^lacZ) cassettes (Lambertsen, Sternberg and Molin, 2004; Hall *et al.*, 2015) in order to distinguish between strains in mixed culture when plated onto Kings Medium B agar (King, Ward and Raney, 1954) (10 g glycerol, 20g proteose peptone no. 3, 1.5g K2HPO4•3H2O, 1.5g MgSO4•7H2O, per litre) supplemented with X-gal (50 μg ml^-1^). All experiments were conducted in 6ml KB broth in 30ml microcosms shaking at 180rpm and incubated at 28°C.

*Selection experiment*

36 independent overnight cultures of Hg^R^ and Hg^S^ strains were mixed at a 1:1 ratio and 60µl (~10^9^ cells ml^-1^) were used to inoculate treatment microcosms. Of the Hg^R^ populations, 18 encoded Hg^R^ on their chromosome and 18 Hg^R^ on the plasmid pQBR57. Six replicate populations were established for each of the three mercury treatments (0, 20 and 40 µM HgCl_2_) and propagated by 1% serial transfer every 24 hours for a total of 8 transfers. Population numbers were determined by diluting and plating onto KB agar + 50ug ml^-1^ X-gal every two transfers. Plasmid prevalence was determined by replica plating agar plates onto KB agar + 100µM HgCl_2_ + 50ug ml^-1^ X-gal which allowed tracking of the plasmid through both donor and recipient populations.

*Competitive fitness assays*

18 independent overnight cultures of mercury resistant (Hg^R^) and mercury sensitive (Hg^S^) strains were mixed at a 1:1 ratio and 60µl (~10^9^ cells ml^-1^) were used to inoculate treatment microcosms. Six replicate populations were established for each of the three mercury treatments (0, 20 and 40 µM HgCl_2_); 3 replicates used Gm^R^ as the reference marker and 3 used the Sm^R^lacZ marker as the reference to control for marker effects. Population numbers were determined by diluting and plating onto KB agar + 50ug/ml X-gal at 0 and 24 hours. Fitness was estimated from these counts as the ratio of Malthusian parameters (W = (ln(test_end_/test_start_)/ (ln(reference_end_/reference_start_)) (Lenski *et al.*, 1991). Based on known estimates of the conjugation rate of pQBR57 in *P. fluorescens* (Hall *et al.*, 2015) conjugation of the plasmid into recipient cells is likely to be minimal across the 24 hours (transconjugants make up ~ 5% of the total population) and thus conjugation has little impact on the fitness estimates.

*MIC measurements*

To measure the minimum inhibitory concentration (MIC) of HgCl_2_ cultures were grown over night until stationary phase in 6ml KB broth. The saturated cultures were then diluted into 96 well plates to an initial density of ~10^3^ cells ml^-1^. Cultures were grown for 24 hours with OD_600_ measured at the end point. The MIC was defined as the lowest concentration which completely inhibited bacterial growth.

*Statistical analysis*

All analyses were conducted in R statistical package version 3.3.2 (R Foundation for Statistical Computing).To assess the effect of gene mobility on the maintenance of Hg^R^, the endpoint proportion of Hg^R^ was analysed using a generalized linear model with gene mobility as a fixed effect. A quasibinomial distribution was used to account for over-dispersion within the data. The model was constructed using only populations subjected to 0 µM HgCl_2_ as populations which had been subjected to the 20 and 40 µM HgCl_2_ treatments did not display adequate variance (i.e. Hg^R^ was fixed at 1 across the majority of populations) within the data and therefore were not suitable for analysis using a GLM. To assess the effect of mercury on conjugative plasmid transfer, the endpoint proportion of transconjugants was analysed using mercury treatment as a fixed effect. Again, a quasibinomial distribution was used to account for over-dispersion within the data. For the fitness assays the competitive fitness of plasmid bearers compared to plasmid-frees was analysed using an ANOVA with mercury treatment as a fixed effect.

**REFERENCES**

Hall, J. P. J., Harrison, E., Lilley, A. K., Paterson, S., Spiers, A. J. and Brockhurst, M. A. (2015) ‘Environmentally co-occurring mercury resistance plasmids are genetically and phenotypically diverse and confer variable context-dependent fitness effects’, *Environmental Microbiology*, 17(12), pp. 5008–5022.

Heckman, K. L. and Pease, L. R. (2007) ‘Gene splicing and mutagenesis by PCR-driven overlap extension’, *Nature Protocols*, 2(4), pp. 924–932.

King, E. O., Ward, M. K. and Raney, D. E. (1954) ‘Two simple media for the demonstration of pyocyanin and fluorescin.’, *The Journal of laboratory and clinical medicine*, pp. 301–307.

Lambertsen, L., Sternberg, C. and Molin, S. (2004) ‘Mini-Tn7 transposons for site-specific tagging of bacteria with fluorescent proteins’, *Environmental Microbiology*. Blackwell Science Ltd, 6(7), pp. 726–732.

Lenski, R. E., Rose, M. R., Simpson, S. C. and Tadler, S. C. (1991) ‘Long-Term Experimental Evolution in Escherichia coli. I. Adaptation and Divergence During 2,000 Generations’, *The American Naturalist*, 138(6), pp. 1315–1341.

Lilley, A. K. and Bailey, M. J. (1997) ‘The acquisition of indigenous plasmids by a genetically marked pseudomonad population colonizing the sugar beet phytosphere is related to local environmental conditions.’, *Applied and environmental microbiology*, 63(4), pp. 1577–83.

R Development Core Team (2016) ‘R: A language and environment for statistical computing’. Vienna, Austria: R Foundation for Statistical Computing.

Rainey, P. B. and Bailey, M. J. (1996) ‘Physical and genetic map of the Pseudomonas fluorescens SBW25 chromosome’, *Molecular Microbiology*, 19(3), pp. 521–533.

Simonsen, L., Gordon, D. M., Stewart, F. M. and Levin, B. R. (1990) ‘Estimating the rate of plasmid transfer: an end-point method.’, *Journal of general microbiology*, 136(11), pp. 2319–25.

**FIGURES**

**Figure S1. The fitness of plasmid-bearing cells significantly increases with mercury selection. a:** The relative fitness (W) of Hg^R^ plasmid-bearers relative to Hg^S^ plasmid-frees was determined across the three mercury treatments (0, 20 and 40 µM HgCl_2_). Points represent means ± SE. A value of 1 indicates equal competitive fitness. **b.** Relative fitness extracted from data presented in Figure 1. Fitness calculated as selection rates between 0 and 48 hours (r=(ln(test_end_/test_start_)- ln(reference_end_/reference_start_))/day) (Lenski *et al.*, 1991). Points represent replicate populations (n = 6). A value of 0 indicates equal competitive fitness. Both chromosome and plasmid-encoded *mer* show increased relative fitness with increasing mercury selection.

**Figure S2. Chromosomal and plasmid-encoded Hg^R^ genes provide equivalent levels of resistance.** Cell density (OD_600_) of SBW25 with chromosomal or plasmid-encoded *mer* as a function of mercury concentration after 24 hours growth in monoculture. Points represent means ± SE of four replicate populations (triangles and circles represent chromosome and plasmid encoded *mer* respectively. Area shaded in grey shows the sub-MIC selective window.
