# Supplementary figures and images for: Gene mobility promotes the spread of resistance in bacterial populations
Source: ISME J. 2017 Mar 31;11(8):1930–2. doi: 10.1038/ismej.2017.42 (PMC5496671; doi:10.1038/ismej.2017.42)

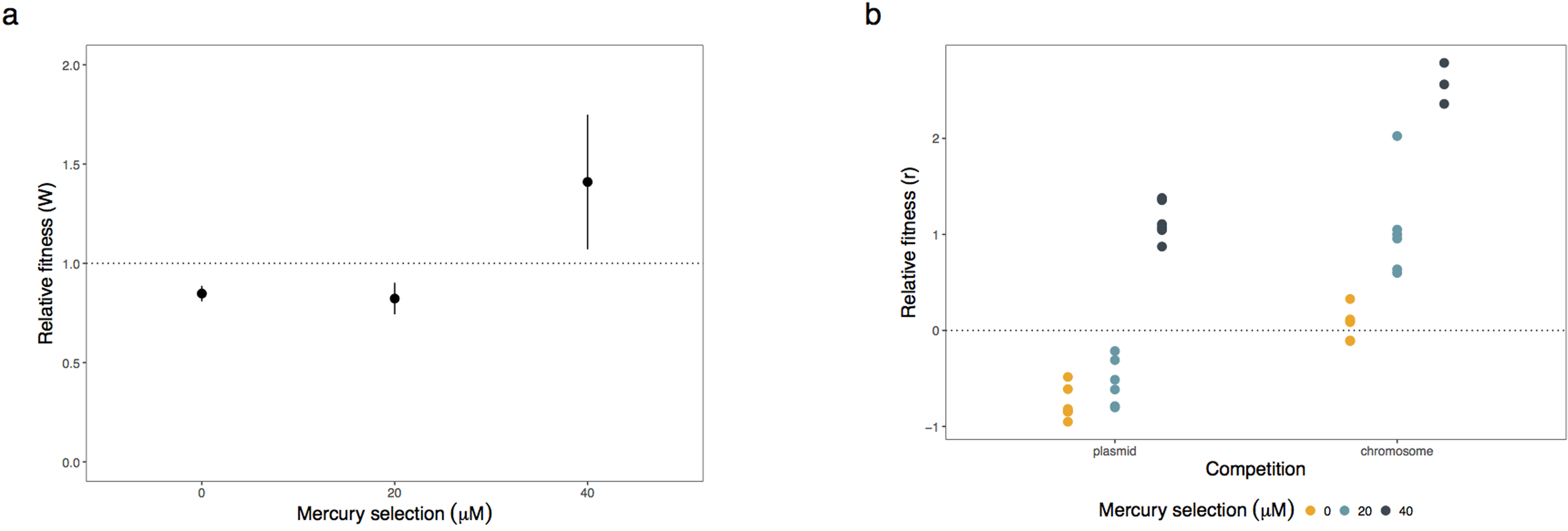

Supplement: Supplementary Figure S1 [file ismej201742x2.tif]

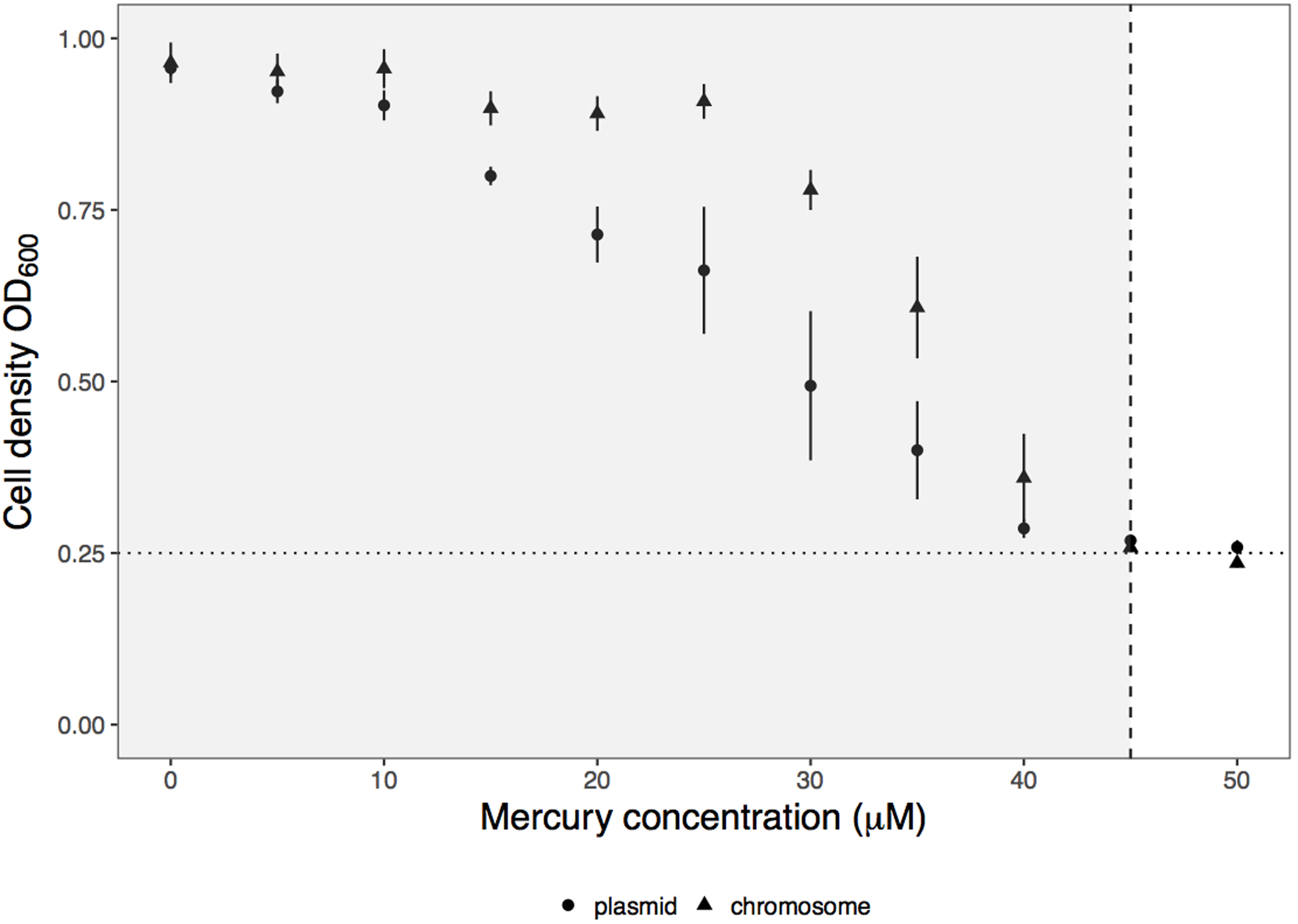

Supplement: Supplementary Figure S2 [file ismej201742x3.tif]
